# A36 NON-PSYCHOTROPIC PHYTOCANNABINOIDS ATTENUATE VISCERAL HYPERSENSITIVITY IN DEXTRAN SULFATE SODIUM (DSS)-INDUCED COLITIS

**DOI:** 10.1093/jcag/gwab049.035

**Published:** 2022-02-21

**Authors:** K Svendsen, M Defaye, K A Sharkey, C Altier

**Affiliations:** University of Calgary Cumming School of Medicine, Calgary, AB, Canada

## Abstract

**Background:**

The inflammatory bowel diseases (IBD), Crohn’s disease and ulcerative colitis, are complex chronic diseases that affect an increasing proportion of the population. Abdominal pain is a major clinical symptom, but current treatments are limited and a source of frustration for patients, many of whom seek alternatives such as cannabis. Cannabis contains many compounds with therapeutic potential that do not have the prohibitive psychotropic effects of tetrahydrocannabinol. These non-psychotropic cannabinoids (npCBs) have a variety of effects including analgesia and anti-inflammatory actions and show potentiating effects when administered in combination. The range of actions of these compounds potentially allows for their development as novel therapeutics for treatment of pain in IBD.

**Aims:**

To investigate the analgesic effects of cannabichromene (CBC), cannabidiol (CBD), cannabidivarin (CBDV), and cannabigerol (CBG), individually and in combination, in the treatment of colitis-evoked visceral hypersensitivity.

**Methods:**

The analgesic effects of the npCBs were investigated in an acute dextran sodium sulfate model of colitis. Abdominal pain was quantified by electromyographic recordings of the reflexive contraction of the external oblique muscles in response to colorectal distension. Activation of the spinal cord was assessed using immunohistochemistry for the neuronal activity marker c-Fos in neurons of the spinal dorsal horn.

**Results:**

CBD reduced pain responses in the functional assay and spinal cord c-Fos activity in a dose-dependent manner. A single intraperitoneal injection of 10 mg/kg, 30 minutes prior to application of the noxious stimulus, reduced pain responses to the level of non-DSS treated control animals. CBDV, and CBG were found to be ineffective in either assay at doses of 1, 5, and 10 mg/kg.

**Conclusions:**

These results suggest CBD may be a promising therapeutic agent in the treatment of colitis–induced visceral hypersensitivity with rapid translational value due to the legalization of cannabis and rapidly growing cannabis industry in Canada. Additionally, CBDV, CBC, and CBG will be further investigated for their analgesic effects and any potentiating effects from administration of multiple npCBs examined.

**Funding Agencies:**

Alberta Innovates

